# The impact of radiosensitivity on clinical outcomes of spinal metastases treated with stereotactic body radiotherapy

**DOI:** 10.1002/cam4.6019

**Published:** 2023-05-10

**Authors:** Lanlan Guo, Qingqing Xu, Lixin Ke, Ziwei Wu, Ziyi Zeng, Lei Chen, Yuanyuan Chen, Lixia Lu

**Affiliations:** ^1^ Sun Yat‐sen University Cancer Center, State Key Laboratory of Oncology in South China, Collaborative Innovation Center for Cancer Medicine Guangdong Key Laboratory of Nasopharyngeal Carcinoma Diagnosis and Therapy Guangzhou People's Republic of China; ^2^ Department of Radiation Oncology Sun Yat‐sen University Cancer Center Guangzhou China; ^3^ Department of Liver Surgery, The First Affiliated Hospital Sun Yat‐sen University Guangzhou China

**Keywords:** local control, spinal metastases, stereotactic body radiotherapy

## Abstract

**Background:**

To evaluate the impact of radiosensitivity on outcomes of spinal metastases treated with stereotactic body radiotherapy (SBRT) and identify the correlated prognostic factors.

**Methods:**

The authors retrospectively reviewed the records of all patients who underwent SBRT with no prior radiation for spinal metastases between October 2015 and October 2020 at Sun Yat‐sen University Cancer Center. On the basis of radiosensitivity, patients were divided into two groups—radiosensitive and radioresistant. The endpoints included local control (LC), overall survival (OS), pain relief, and time to pain relief.

**Results:**

A total of 259 (82.5%) patients with 451 lesions were assessable with a median follow‐up time of 10.53 months. The 1‐, 2‐, and 3‐year OS rates were 59%, 52%, and 44%, respectively. The median survival was 33.17 months. Higher Karnofsky Performance Scale score and shorter time to diagnosis of spinal metastases from primary cancer at consult predicted for better OS (*p* = 0.02 and *p* < 0.001, respectively). The presence of other metastases (*p* = 0.04) and pain at enrollment assessed by the Brief Pain Inventory predicted for worse OS (*p* = 0.01). The 6‐, 12‐, and 24‐month LC rates were 88%, 86%, and 82%, respectively. Younger age was identified for better LC and pain relief (*p* < 0.001 and *p* = 0.04, respectively). There was no variable independently associated with time to pain relief. As for toxicity, no Grade ≥3 toxicity was observed.

**Conclusions:**

Regardless of radiosensitivity, SBRT is feasible and appears to be an effective treatment paradigm for patients with spinal metastases, with limited accepted toxicities.

## 1 INTRODUCTION

Following lung and liver metastases, bone metastases are regarded as the most common tumor metastases, mostly in the spine.^1^, ^2^ According to the estimation, up to 40% of cancer patients will develop spinal metastases during their lifetime.^3^ Over 70% of spinal metastases are situated in the thoracic or lumbar spine; cervical spine metastases and sacrum are included in 4%–18% and 5% of the sample in recent series, respectively.^4^, ^5^ More than 10% of cancer patients with spinal metastases will develop metastatic epidural spinal cord compression (ESCC).^6^, ^7^ The occurrence of spinal metastases can lead to substantial morbidity. For patients with spinal metastases, the conventional treatment paradigm includes chemotherapy, targeted therapy, surgery, radiotherapy, and immunotherapy, which need multidisciplinary cooperation. Additionally, with the development of surgical techniques and implementations, more and more clinicians choose to treat patients with adjuvant radiation therapy. In the meanwhile, stereotactic body radiotherapy (SBRT) has emerged as an attractive alternative for spinal metastases. In particular, SBRT can deliver high doses of radiation to the target lesions with high accuracy, while sparing the critical organs at risk (OARs) to a great extent. However, it might be due to careful patient selection that SBRT might develop further in the treatment of spinal metastases. Selecting patients treated with spinal SBRT is of great significance, which can avoid the catastrophic consequences of failure. For carefully selected patients, SBRT could be a standard treatment modality.

The goal of treatment for patients with spinal metastases remains palliative, in order to prevent the progression or retreatment, delaying it at least. The definition of pain flare was temporary worsening of pain in the treated site, which maybe a potential side effect of palliative radiotherapy for symptomatic bone metastases.^8^ Therefore, a better understanding of long‐term efficacy and safety about these treatment modalities is essential. According to previous studies,^9^, ^10^, ^11^, ^12^, ^13^, ^14^, ^15^, ^16^ radioresistant histological types included renal cell carcinoma (RCC), melanoma, and sarcoma, which indicate a poor local tumor control treated with conventional external beam radiotherapy (cEBRT); radiosensitive histological types included lymphoma, seminoma, myeloma, prostate cancer, and breast cancer. A study^17^ exploring the impact of histology and dose on local control (LC) of spinal metastases treated with stereotactic radiosurgery (SRS) demonstrated that high‐dose single‐session SRS can provide durable tumor control, irrespective of the histology or tumor size. However, there was no statistical analysis in histology. We conducted this study to further evaluate the prognosis and adverse effects of SBRT for spinal metastases with different radiosensitivity. The purpose of this study is to further promote the indications of SBRT and lay a foundation for clinical decision‐making. Based on this, it might provide a reference for further prospective clinical trials.

Therefore, this study aimed to assess the influence of tumor radiosensitivity on the effect of SBRT to patients with spinal metastases, and attempt to elucidate indications for patient selection. Further categorizing tumors with different radiosensitivity (radiosensitive, radioresistant),^10^, ^14^, ^17^, ^18^, ^19^, ^20^ of the classification of our study, radiosensitive carcinomas included small cell lung cancer, nasopharyngeal carcinoma, breast cancer, and prostate cancer. Also, radioresistant carcinomas included liver cancer, melanoma, sarcoma, and pancreatic cancer. We specifically examined pain relief, time to pain relief, durable LC, overall survival (OS), and toxicities after SBRT for spinal metastases.

## 2 METHODS

### 2.1 Patient population and selection criteria

We retrospectively reviewed a consecutive series of 547 lesions in 314 patients who were treated with SBRT for spinal metastases at Sun Yat‐sen University Cancer Center between October 2015 and October 2020, and gathered outcomes until December 2020. All patients had histologically proven primary cancer diagnosis. Spinal metastases were diagnosed by CT and/or MRI and/or PET‐CT. Institutional review board (IRB) approval was obtained at the Sun Yat‐sen University Cancer Center, Guangzhou, China.

Eligibility criteria included: age >18 years; a pathological diagnosis of primary cancer; oligometastasis (five or fewer radiographically apparent metastases.); KPS score of ≥70; lesions treated with radiographic or clinical follow‐up; a maximum of three separate sites of treatment, up to two contiguous levels within one radiation field. Patients were ineligible if they had acute spinal cord compression or the presence of overt spinal instability.

### 2.2 SBRT treatment

SBRT was performed using intensity‐modulated radiation therapy planning or other dose painting techniques. Unless contraindicated, all patients underwent 3 mm slice thickness contrast‐enhanced CT and/or MRI simulation scanning. Patients received radiation in one to six fractions with a 6–8 MV X‐ray beam applied using the Elekta Precise Treatment System (Elekta AB) or the Elekta Versa HD System (Elekta AB). The contouring guidelines for the targets and organs‐at‐risk, dose delivering and dose restriction of spinal metastases treated with SBRT in accordance with previous studies^21^, ^22^, ^23^, ^24^, ^25^ and RTOG 0631 protocol.^26^ The prescription doses in the study were included. Also, the delivered doses in the study meet the criteria of the hypofractionated radiation therapy.

### 2.3 Outcomes assessment

LC was defined as no progression within the treated vertebral body level on following CT or MRI. Vertebral compression fracture (VCF) was defined as de novo and progressive preexisting fractures. Toxicity was graded according to the Common Terminology Criteria for Adverse Events (CTCAE) version 5.0.^27^ Pain relief included complete pain relief and partial pain relief. Complete pain relief is defined as a pain score of 0 at the lesion site is 0 at 3 months after treatment, with based on no increase in anesthetic analgesics. Partial pain relief is defined as a reduction of ≥3 points in the pain score at the lesion site (i.e., an improvement of at least 3 points from baseline Brief Pain Inventory [BPI] score), no increase in the pain score for other treatments, and no need to increase the dose of narcotic pain drugs. However, even if the pain score is decreased by ≥3 points, patients who require an increase in the dose of narcotic analgesics are not considered to achieve partial pain relief. For assessment of pain palliation, the 4‐point verbal rating scale (VRS‐4)^28^ was administered, along with a categorical scale representing different levels of pain intensity: “none,” “mild,” “moderate,” “severe.” Epidural extent was graded utilizing criteria by Bilsky et al.^29^ Usually, the word “oligometastases” is defined as ≤5 radiographic metastases.

### 2.4 Data collection

A retrospective review of the hospital records and radiographic studies of these patients was performed. Data on each patient including age, sex, date of initial diagnosis of primary cancer, histology of primary cancer, date of diagnosis of first spinal metastasis, location of the spinal metastasis (single vertebral segment, only thoracic vertebra or lumbar vertebra), radiation sites, other metastases (metastases of all sites other than treated bone metastases), further radiotherapy to other sites, the presence of paraspinal extension, further systemic treatment, number of the spinal metastasis, previous treatments (including surgery, chemotherapy, and radiation therapy [RT]) and KPS score, pre‐SBRT pain as assessed by the BPI (when available), ESCC grade pre‐SBRT, and SBRT information (including RT dose, and number of RT fractions delivered) were gathered.

### 2.5 Statistical analysis

Both continuous and categorical variables were summarized. The Kaplan–Meier product‐limit method was used to estimate OS, and survival curves were compared using the log‐rank test. Factors associated with OS after SBRT were assessed using univariate and multivariate Cox proportional hazards analyses, as well as LC. Hazard ratios (HRs) and their corresponding 95% confidence intervals (CIs) were computed. Factors associated with pain palliation and the incidence of SBRT complications were assessed using univariate and multivariate logistic regression analyses. Factors associated with the time of pain palliation were assessed using linear regression. All mean values are presented as mean ± standard error. All *p*‐values were two‐sided. Here, a *p* < 0.05 was considered statically significant. Statistical analysis was performed using SPSS software (version 26.0, IBM).

## 3 RESULTS

### 3.1 Patient demographics

During the study period, 314 patients treated with SBRT were included between October 2015 and October 2020, and 55 patients have been lost to follow‐up. The demographic, tumor, and treatment characteristics of 259 patients with 451 lesions are summarized in Table 1. One hundred eighty‐four patients (71.0%) were male, 75 (29.0%) were female. The median age was 57 years (range, 19–89 years). The median KPS score was 90 (range, 70–100). The median follow‐up for all patients was 10.53 months (range, 0.33–59.03 months). The minimal follow‐up time is 0.33 month.

**TABLE 1:** Baseline patient demographic, tumor, and treatment characteristics.

Characteristic	Radiosensitive		Radioresistant		Total	
	No. of Patients	%	No. of Patients	%	No. of Patients	%
All patients	76	29.3	183	70.7	259 of 314	82.5
Sex						
Male	64	84.2	120	65.6	184	71.0
Female	12	15.8	63	34.4	75	29.0
Age at time of SBRT (year)						
Range	31–89		19–86		19–89	
Median	67		55		57	
Other metastases						
Yes	13	17.1	43	23.5	56	21.6
No	63	82.9	140	76.5	203	78.4
Surgery						
Yes	5	6.6	35	19.1	40	15.4
No	71	93.4	148	80.9	219	84.6
Karnofsky performance status						
Range	70–95		70–100		70–100	
Median	90		90		90	
Spine location/level						
Cervical	8	10.5	19	10.4	27	10.4
Thoracic	46	60.5	83	45.4	129	49.8
Lumbar	18	23.7	68	37.2	86	33.2
Sacral	4	5.3	13	7.1	17	6.6
Bilsky epidural Grade						
0	60	78.9	126	68.9	186	71.8
1a	1	1.3	7	3.8	8	3.1
1b	7	9.2	24	13.1	31	12.0
1c	4	5.3	5	2.7	9	3.5
2	4	5.3	21	11.5	25	9.6
Number of lesions						
Range	1–4		1–7		1–7	
Median	1		1		1	
Paraspinal extension						
Yes	14	18.4	72	39.3	86	66.8
No	62	81.6	111	60.7	173	33.2
Systemic therapy						
Chemotherapy	72	94.7	90	49.2	162	62.5
Bisphosphonate therapy	55	72.4	101	55.2	156	60.2
Other radiation						
Yes	55	72.4	41	22.4	73	28.2
No	21	27.6	142	77.6	186	71.8
Total dose (Gy)						
Range	8–45		8–48		8–48	
Median	27		30		30	
Fractions						
Range	1–6		1–6		1–6	
Median	5		5		5	
Time to diagnosis of spinal metastases from primary cancer						
Range	0–177.53		0–181.37		0–181.37	
Median	9.69		5.87		6.33	

Among all treated lesions, 27 (10.4%) were cervical, 129 (49.8%) thoracic, 86 (33.2%) lumbar, and 17 (6.6%) sacral. There were 258 patients defined as oligometastases. The most common lesions treated were metastatic tumors of the lung (*n* = 66), followed by prostate cancer (*n* = 66) and others. The ESCC grades were 0 in 186 (71.8%), 1a in 8 (3.1%), 1b in 31 (12.0%), 1c in 9 (3.5%), and 2 in 25 (9.6%). Of the 24 patients with epidural disease compressing the spinal cord (Bilsky Grade 2), 12 underwent surgery. Because of the medical comorbidities and intolerance for surgery, the other 12 patients were treated with SBRT alone. The spinal metastases with paraspinal extension were 82 (40.59%). One hundred sixty‐two (62.5%) patients experienced prior chemotherapy, and 156 (60.2%) prior bisphosphonate therapy. Patients treated with radiation therapy on other sites were 73 (28.2%). In this consecutive series, 137 (30.4%) lesions were from radiosensitive tumors, 341 (69.6%) from radioresistant malignancies.

The median interval from the initial diagnosis of primary cancer to the diagnosis of spinal metastases was 6.33 months (range, 0–181.37 months). Of the 451 lesions, 40 (15.4%) were treated with SBRT postoperatively. The median total dose delivered was 30 Gy (range, 8–48 Gy), with a median fraction number of 5 (range, 1–6). The median biologically effective dose delivered was 48.0 Gy (range, 14.4–86.4 Gy).

### 3.2 Local control

**FIGURE 1:**
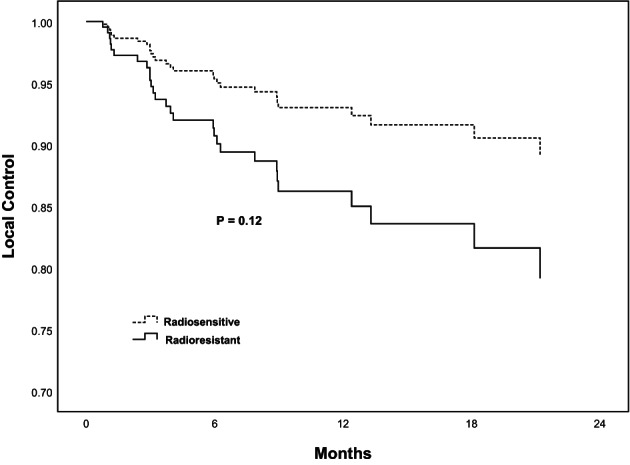
Local control for radiosensitive versus radioresistant spinal metastases of 259 patients.

The 6‐, 12‐, and 24‐month LC rates were 88%, 86%, and 82%, respectively. On univariate analysis, age was the only factor associated with LC rate (*p* < 0.001). There was no statistical significance in radiosensitivity (*p* = 0.12) (Figure 1). On multivariate analysis, age and dose were significant factors associated with LC rate (*p* < 0.001 and *p* = 0.02) (Table 2).

**TABLE 2:** Univariate and multivariate analysis of local control of 259 patients.

Variable	Univariate hazard ratio	*p*‐Value	Multivariate hazard ratio	*p*‐Value
Sex	1.02 (0.43–2.41)	0.96	1.63 (0.64–4.14)	0.30
Age	0.95 (0.93–0.98)	<0.001	0.93 (0.90–0.97)	<0.001
Treating site	0.81 (0.49–1.34)	0.40	0.71 (0.39–1.31)	0.28
Paraspinal extension	1.74 (0.82–3.70)	0.15	1.53 (0.63–3.73)	0.35
KPS score at consult	0.99 (0.93–1.06)	0.78	1.01 (0.95–1.08)	0.68
Radiosensitivity	2.05 (0.82–5.09)	0.12	1.48 (0.47–4.67)	0.50
Number of lesions	0.68 (0.42–1.10)	0.11	0.78 (0.46–1.30)	0.34
Dose	1.00 (1.00–1.00)	0.69	1.00 (1.00–1.00)	0.02
Fractions	0.92 (0.70–1.20)	0.54	1.53 (0.93–2.51)	0.10
Time to diagnosis of spinal metastases from primary cancer	0.98 (0.96–1.00)	0.10	0.99 (0.97–1.01)	0.43
Other metastases (Yes/No)	1.47 (0.56–3.87)	0.44	1.78 (0.65–4.90)	0.26
Bilsky grade	1.18 (0.94–1.48)	0.15	1.16 (0.89–1.52)	0.27
Surgery (Yes/No)	1.80 (0.76–4.23)	0.18	0.75 (0.26–2.15)	0.59
Bisphosphonate (Yes/No)	0.75 (0.36–1.58)	0.45	0.90 (0.39–2.08)	0.81
Other radiation (Yes/No)	0.64 (0.26–1.58)	0.33	0.98 (0.35–2.73)	0.97
Chemotherapy (Yes/No)	0.60 (0.28–1.27)	0.18	0.52 (0.21–1.29)	0.16
Pain	0.94 (0.44–2.00)	0.87	0.96 (0.42–2.17)	0.92

### 3.3 Overall survival

**FIGURE 2:**
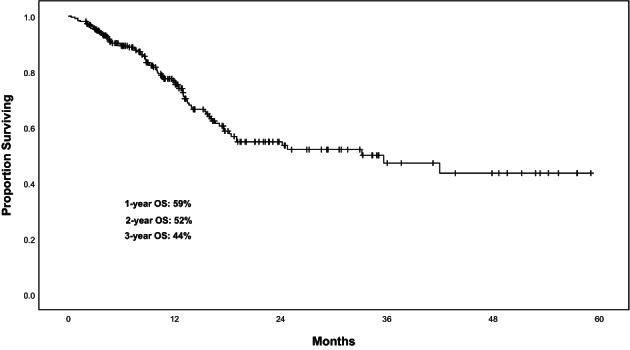
Kaplan–Meier chart showing overall survival after SBRT in spinal metastases.

**FIGURE 3:**
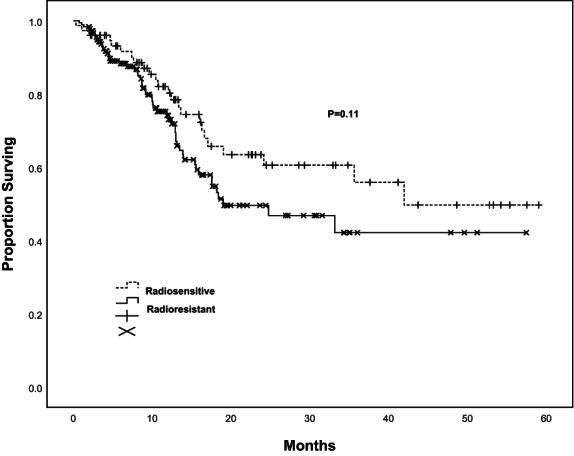
Overall survival for radiosensitive versus radioresistant spinal metastases of 259 patients.

**FIGURE 4:**
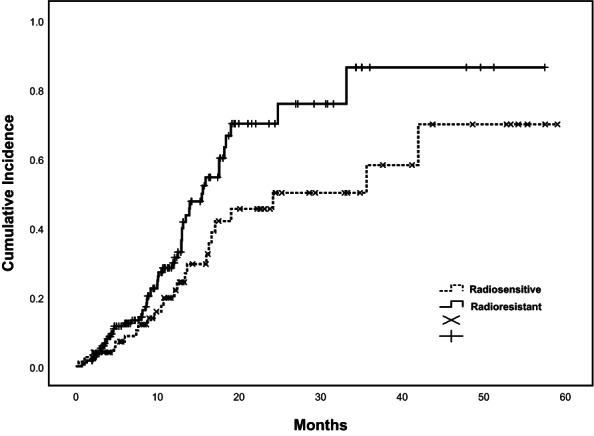
Cumulative incidence for radiosensitive versus radioresistant spinal metastases.

The OS rates at 1, 2, and 3 years were 59%, 52%, and 44%, respectively (Figure 2). One hundred fourteen patients were still followed up at the 3‐year timepoint. The mean survival was 34.45 months (95% CI, 30.31–38.59 months). The median survival was 33.17 months. There was no statistically significant difference in radiosensitivity associated with OS (*p* = 0.11) (Figure 3). The cumulative incidence for radiosensitive and radioresistant spinal metastases is shown in Figure 4. On univariate analysis, the factors that significantly affected OS were KPS score (*p* = 0.02), the presence of other metastases (*p* = 0.04), treating site (*p* = 0.02), and the presence of pain at enrollment (*p* < 0.001). On multivariate analysis, KPS score (*p* = 0.04), treating site (*p* = 0.04), the presence of pain (*p* = 0.02), and other metastases (*p* = 0.02) were significant factors of worse OS (Table 3).

**TABLE 3:** Univariate and multivariate analysis of overall survival of 259 patients.

Variable	Univariate hazard ratio	*p*‐Value	Multivariate hazard ratio	*p*‐Value
Sex	0.82 (0.49–1.35)	0.43	0.99 (0.55–1.77)	0.97
Age	1.00 (0.99–1.02)	0.93	1.02 (0.99–1.04)	0.15
Treating site	1.40 (1.05–1.85)	0.02	1.37 (1.02–1.86)	0.04
Paraspinal extension	1.03 (0.64–1.67)	0.90	1.05 (0.60–1.85)	0.85
KPS score at consult	0.96 (0.92–0.99)	0.02	0.96 (0.92–1.00)	0.04
Radiosensitivity	1.48 (0.91–2.40)	0.11	0.97 (0.51–1.86)	0.93
Number of lesions	1.08 (0.87–1.35)	0.48	0.98 (0.77–1.24)	0.85
Dose	1.00 (1.00–1.00)	0.46	1.00 (1.00–1.00)	0.78
Fractions	1.08 (0.89–1.30)	0.43	1.11 (0.84–1.47)	0.47
Time to diagnosis of spinal metastases from primary cancer	1.00 (0.99–1.01)	0.55	1.00 (0.99–1.01)	0.52
Other metastases (Yes/No)	1.91 (1.03–3.54)	0.04	2.16 (1.12–4.15)	0.02
Bilsky grade	0.91 (0.76–1.08)	0.28	0.89 (0.71–1.10)	0.28
Surgery (Yes/No)	0.73 (0.38–1.42)	0.35	0.80 (0.36–1.78)	0.59
Bisphosphonate (Yes/No)	1.34 (0.84–2.15)	0.22	1.40 (0.85–2.30)	0.19
Other radiation (Yes/No)	0.95 (0.59–1.53)	0.82	1.05 (0.61–1.82)	0.85
Chemotherapy (Yes/No)	1.01 (0.63–1.63)	0.96	1.24 (0.72–2.15)	0.43
Pain (Yes/No)	2.15 (1.37–3.37)	<0.001	1.81 (1.12–2.93)	0.02

### 3.4 Pain relief

Of the 143 patients with baseline pain, 8 patients were lost to follow‐up and 135 patients were assessable. Because of the retrospective characteristic of our study, it was hard to avoid several patients not to keep up with the follow‐up. The range “0” of time to pain relief means that the patients attained pain relief on the day of radiation. Almost at the time of receiving radiation, pain palliation was achieved. We reported a 76.3% (103/135 patients) of pain relief. Univariate analysis identified age (*p* = 0.04) to be prognostic for better pain relief. On multivariate analysis, age retained its significance in terms of pain relief (*p* = 0.04). The others were not significant factors, including treating site, paraspinal extension, KPS score at consult, the number of metastases, dose, fractions, time to spinal metastases, Bilsky grade, and use of surgery. The median time to pain relief was 0.8 months (range, 0–14.67 months). On univariate and multivariate analyses, there were no significant factors with time to pain relief.

### 3.5 Treatment after SBRT

Radiotherapy is considered as a local treatment. Therefore, after SBRT, 314 underwent systemic therapy for primary cancer, including chemotherapy in 129 (41.08%) patients, immunotherapy in 43 (13.69%) patients, and targeted therapy in 105 (33.44%) patients. Obviously, almost these treatments were for primary cancer. In addition, other local treatments were applied, including surgery in 8 (2.55%) patients and radiation for other sites in 66 (21.0%) patients. Meanwhile, 156 patients (49.68%) were receiving bisphosphonate. Bisphosphonates were mainly used in reducing the incidence of bone disease, bone pain, and fracture.

### 3.6 Toxicity

All patients tolerated SBRT well with minimal complications or toxicity, with no Grade ≥3 toxicities observed in any patient. And there were no cases of myelopathy or radiculopathy. Overall, 26 (8.28%) patients experienced VCFs. Of these patients, two were surgically stabilized before SBRT, with 24 VCFs occurring after SBRT in the nonsurgical sites. Twenty‐one of 26 were de novo VCFs and others were the progression of an existing VCF. On univariate analysis, the factor that significantly affected the rate of VCF was use of bisphosphonate (*p* = 0.045). There was no statistically significant difference for radiosensitivity (*p* = 0.39). On multivariate analysis, the use of bisphosphonate was a predictor for the occurrence of VCF (*p* = 0.02). Two patients experienced pain flare after SBRT that required hospitalization. Grade 1/2 toxicity consisted of fatigue during treatment for nine patients, and one patient had decreasing smelling after SBRT not requiring treatment. No patient had persistent or worsening neurological symptoms.

## 4 DISCUSSION

In 1995, spinal radiosurgery was first applied to patients with spinal metastases, who demonstrated radiographic recurrence or progression after treatment with cEBRT.^30^ When spinal metastases are presented with symptomatic VCF, mechanical instability, and acute ESCC, surgery should be considered in many cases.^31^ For patients not requiring surgery or unbearable to surgery, cEBRT is taken into consideration (low‐ to intermediate‐dose in 1 or more fractions).^18^ With imaging and treatment technology developing, SBRT has increasingly established its role in the treatment for spinal metastases. Selecting patients treated with spinal SBRT is of great significance, which can avoid the catastrophic consequences of failure. For carefully selected patients, SBRT could be a standard treatment modality.

In our study, of patients with radiographic follow‐up, 1‐year LC rate was 86%. The result was consistent with several previous analysis.^32^, ^33^, ^34^, ^35^, ^36^ In addition, 6‐month LC rate was 88%. Another study^37^ reported that 6‐ and 9‐month LC rates were 86% and 86%, respectively. The rates were consistent with our result. The 2‐year LC rate ranged from 73% to 83.9%.^32^, ^33^, ^36^ The 2‐year LC rate was 82% in our study. With regard to different histologies, LC rates varied from 80% to 100%, while no statistically significant difference was found.^17^ In some degree, different histologies represent different biological behaviors, thus lead to different radiosensitivity. After our analysis, there was no statistically significance in radiosensitivity (*p* = 0.12). According to above studies, we can think that SBRT could provide durable local tumor control regardless of radiosensitivity. Of the 28 patients regarded as local failure, liver cancer and renal cell cancer take up 21.4%, respectively. As for LC, we reported that age was a statistically significant factor (*p* < 0.001). Younger seemed to have a better local tumor control. In future patient selection, age could be taken into consideration.

We also paid attention to the survival outcomes. We reported that the OS rates at 1‐ and 2‐year were 59% and 52%, respectively. These results are consistent with prior reports in the literature.^33^, ^34^, ^36^, ^38^ Compared with previous results,^17^, ^33^, ^34^, ^38^, ^39^ there were similar median and mean survival periods demonstrated in our study, 33.17 and 34.45 months (95% CI, 30.31–38.59 months), respectively. In some degree, it suggested appropriate patient selection via definite inclusion and exclusion criteria.

KPS score was used to evaluate general condition of patients. Obviously, higher KPS score, and better general condition. It is regarded as a predictor for OS (*p* = 0.02). With better general condition, patients with spinal metastases would accomplish the treatment and gain the survival benefit from SBRT. Moreover, the presence of other metastases and pain at enrollment were significant factors for OS (*p* = 0.04, *p* < 0.01). In those who experienced pain at enrollment, achieving pain relief could be a predictor for better OS (*p* < 0.01). As is known to us, for bone metastases, including spinal metastases, the most common symptom was pain. Therefore, with regard to patients with baseline pain, timely and optimal analgesia treatment modality was critical before SBRT. Of course, both analgesics and radiotherapy produced analgesic effects. But there was no denying that we did see better OS among patients who had attained pain relief. In the meanwhile, after the diagnosis of primary cancer, regular physical examination is essential in case of the occurrence of spinal metastases (*p* < 0.04). It demonstrated that treating site was a significant factor for OS (*p* = 0.02). Overall, it indicated that cervical vertebrae had a better OS (27 patients), followed by lumbar vertebrae (86 patients), thoracic vertebrae (129 patients), and sacral vertebrae (17 patients). It may be because sacral vertebrae are adjacent to ilium. Therefore, some patients developed myelosuppression after radiation therapy. Through the statistical analysis, there was no significant difference found in radiosensitivity with LC (*p* = 0.12) and OS (*p* = 0.11). It could be predicted that spinal SBRT provides a satisfying LC and OS, regardless of radiosensitivity.

For these patients, SBRT aims to achieve the pain palliation and delay or even prevent the retreatment. We reported 76.3% of pain relief rate, similar to previous reported rates.^32^, ^34^, ^40^, ^41^ Univariate and multivariate analyses identified age (*p* = 0.04) to be a prognostic factor for pain relief. Younger age could achieve better pain relief. In a way, it may be associated with faster cell proliferation in younger people. When selecting patients to be treated with SBRT, age might be taken into consideration.

To our knowledge, there are no uniformly accepted indications in patient selection for spinal SBRT. The above factors might provide instructions in selecting patients. Moreover, the neurologic, oncologic, mechanical, and systemic (NOMS) framework^42^ and the location of disease in the spine, mechanical instability, neurology, oncology, and patient fitness, prognosis and response to prior therapy framework (LMNOP)^43^ might play an important role in patient selection.

The most common toxicity for spinal metastases treated with SBRT was the occurrence of VCF. For patients, VCF would have an influence on the quality of life, such as intolerable bone pain. Hence, fracture prevention is of great importance. As for the toxicity of patients treated with SBRT, we demonstrated a risk of VCF of 8.28%, which is consistent with previous spinal SBRT literatures reporting rates ranging from 5% to 42%.^44^ The use of bisphosphonate (*p* = 0.045) could decrease the risk of VCF. Therefore, it is necessary to take some preventive measures to avoid the occurrence of VCF after SBRT.

Several strengths of this study deserve mention. First, we categorized tumors with different radiosensitivity. No statistically significant difference was found in radiosensitivity with OS, LC, pain relief, and time to pain relief. Our results might provide a reference for future research. Second, in the study, we analyzed the aspects including OS, LC, pain relief rate, and time to pain relief. Obviously, it included and analyzed comprehensive outcomes. Third, without limited in single fraction, we reported one to six fractions; the results were consistent with previous studies. Moreover, to the best of our knowledge, our study was the first to report the outcomes of spinal metastases treated with SBRT in China. There were a number of limitations in this study. Given the retrospectively collected data, selection biases inevitably existed. Furthermore, assessment of pain response was not prospectively collected. The study is limited because of the experience of single institution, which is needed to be validated in other institutions.

## 5 CONCLUSIONS

In conclusion, regardless of radiosensitivity, SBRT is a noninvasive intervention that seems to be viable, safe, and effective for patients with spinal metastases. With minimal accepted toxicities, a good tumor control was achieved. Furthermore, patients can be expected to develop durable LC and pain relief on treated lesions. To confirm late toxicities and define patient selection, further studies with large sample sizes and a longer follow‐up are required.

## AUTHOR CONTRIBUTIONS

**Lanlan Guo:** Data curation (lead); formal analysis (lead); methodology (lead); resources (lead); software (lead); writing – original draft (lead). **Qing‐Qing Xu:** Investigation (equal); methodology (equal); resources (equal); software (equal); writing – original draft (equal). **Lixin Ke:** Conceptualization (equal); methodology (equal); resources (equal); software (equal); writing – original draft (equal). **Ziwei Wu:** Data curation (equal); formal analysis (equal); methodology (equal). **Ziyi Zeng:** Formal analysis (equal); software (equal); validation (equal). **Lei Chen:** Methodology (equal); project administration (equal); supervision (equal). **Yuan‐Yuan Chen:** Methodology (equal); project administration (equal); supervision (equal). **Li‐Xia Lu:** Methodology (equal); project administration (equal); supervision (equal); writing – review and editing (equal).

## CONFLICT OF INTEREST STATEMENT

The authors have no conflict of interest to declare.

## ETHICAL APPROVAL

The study was approved by the ethics committee of Sun Yat‐Sen University Cancer Center (number SL‐B2021‐237‐01).

## Data Availability

The retrospective data used to support the findings of this study are available from the corresponding author upon request.
